# Insomnia as a predictor of treatment outcomes in adolescents receiving concentrated exposure treatment for OCD

**DOI:** 10.1186/s12888-024-06183-3

**Published:** 2024-10-18

**Authors:** Solvei Harila Skjold, Kristen Hagen, Michael G. Wheaton, Håvard Kallestad, Kay Morten Hjelle, Thröstur Björgvinsson, Bjarne Hansen

**Affiliations:** 1 Bergen Center for Brain Plasticity, Haukeland University Hospital, Bergen, Norway; 2 Center for Crisis Psychology, Faculty of Psychology, University of Bergen, Bergen, Norway; 3 Møre and Romsdal Hospital Trust, Molde, Norway; 4 Haukeland University Hospital, OCD-team, Bergen, Norway; 5 Barnard College, Columbia University, New York, USA; 6 Center for OCD and Related Disorders, Massachusetts General Hospital, Boston, USA; 7 Harvard Medical School, Boston, USA; 8 McLean Hospital, Belmont, USA; 9 NTNU, Trondheim, Norway

**Keywords:** OCD, Insomnia, Sleep disturbance, Adolescents, Concentrated treatment, Exposure, B4DT, CBT

## Abstract

**Background:**

Research suggests that individuals with obsessive-compulsive disorder (OCD) frequently experience insomnia. Some previous studies have suggested that insomnia may predict treatment outcomes, but the evidence is limited, especially for adolescents. This study examined the prevalence of insomnia in an adolescent OCD patient sample, explored the correlation between OCD and insomnia, and tested whether levels of insomnia at baseline predict outcomes for adolescent patients receiving the Bergen 4-Day Treatment (B4DT) for OCD.

**Methods:**

Forty-three adolescent OCD patients who received B4DT were selected for this study. Treatment outcome was quantified as change in Children Yale-Brown Obsessive Compulsive Scale (CY-BOCS) scores across time from pre- to posttreatment and 3-month follow-up. Insomnia symptoms were measured by the Bergen Insomnia Scale (BIS). Linear mixed models were used to examine the relationship between the BIS and changes in CY-BOCS scores. We controlled for symptoms of general anxiety disorder measured by the GAD-7 and depression symptoms measured by the PHQ-9.

**Results:**

In this sample, 68.4% of the patients scored above the cutoff for insomnia on the BIS. There was a moderate correlation between baseline CY-BOCS and BIS that did not reach statistical significance (*r* = .32, *p* = .051). High BIS scores before treatment were significantly associated with poorer treatment outcomes, as measured by changes in CY-BOCS over time (*p* = .002). The association between baseline insomnia and change in OCD symptoms remained significant (*p* = .033) while controlling for GAD-7 and PHQ-9.

**Conclusion:**

Insomnia is common among adolescents with OCD, and these data suggest that these patients may be at increased risk for poor treatment outcomes. Future research to explore mechanisms and adjunctive treatments is warranted.

**Trial registration:**

The study was approved by the Regional Committee for Medical and Health Research Ethics of Northern Norway (REK Nord: 2023/606482).

## Introduction

Obsessive-compulsive disorder (OCD) is characterized by obsessions and compulsions that impair daily functioning. The disorder typically develops during childhood or adolescence, as more than 50% of OCD patients report that they had symptom onset during this period [1, 2]. Spontaneous recovery without treatment is rare [3, 4]. However, OCD treatment with exposure and response prevention (ERP) is effective for many patients, either in traditional formats or in brief, intensive or concentrated formats [5–7]. The Bergen 4-Day Treatment (B4DT) is an example of concentrated treatment that is effective in treating OCD [8–12].

Although there is effective OCD treatment that helps many patients recover from their OCD, a sizable minority of patients do not respond to treatment or experience only a partial reduction in symptoms or subsequently relapse [5, 6]. Thus, finding factors that influence treatment outcomes is needed to try to identify patients at risk for poor outcomes and refine treatment to help more patients recover fully. Several studies have tried to identify predictors of outcome for OCD patients to personalize and optimize treatment, but the results are somewhat inconsistent [13–15]. In the NordLOTS study, comorbidity, gender, socioeconomic status, symptom severity, and duration of symptoms did not predict OCD treatment outcomes for children and adolescents with OCD, but age and sleep problems pretreatment did [14, 16]. One study with adult OCD patients (who were defined as difficult-to-treat based on nonresponse to CBT or relapse) found that sleep problems predicted OCD treatment outcome [17]. However, other studies with adult OCD patients receiving B4DT did not find a link between sleep problems and OCD treatment outcomes [18, 19]. Thus, research in this area is scarce and inconclusive.

Sleep disorders and sleep problems include insomnia, obstructive sleep apnea, restless leg syndrome, narcolepsy, and parasomnias [19, 20]. The most common sleep disorder is insomnia disorder [19]. Insomnia is characterized by difficulties initiating or maintaining sleep, accompanied by daytime symptoms such as fatigue, irritation, or lack of concentration, leading to functional impairment [20, 21]. Insomnia can lead to reduced functioning in various areas, which may limit the effectiveness of OCD treatment [22–26]. Research suggests that there is a connection between insomnia and OCD symptoms, although exactly how the two factors affect each other, or what causes what, remains unknown [27–29]. OCD patients frequently have insomnia or sleep disorders [18]. In support of a link between insomnia and OCD, a Swedish population study suggests that the chance of having an insomnia diagnosis or receiving a drug for insomnia is approximately 7 times more likely for OCD patients compared to the general population [30].

Insomnia is common among adolescents in general, although the prevalence numbers vary in different studies as different criteria of insomnia are used [31]. People who are diagnosed with psychiatric diagnoses have more sleep problems than the general population, as several different diagnoses are associated with higher prevalences of sleep problems [32, 33]. The prevalence of insomnia for adolescents with OCD also varies in different studies [27]. A Swedish study of children and adolescents [33] found that 28% of OCD patients and 20% of patients in children and adolescent psychiatric outpatient clinics had sleep problems, while only 5% of school-aged children in general had the same problems. In the NordLOTS study, a large Scandinavian study of CBT for pediatric OCD patients, the prevalence of sleep problems in the youth population with OCD was even higher, where 68% had at least one mild sleep problem [15]. Thus, the prevalence of sleep problems among children and adolescents with OCD seems higher than in the general child and adolescent population.

The need for sleep and sleep problems may differ at different developmental stages, so sleep problems among children or adults may be different from sleep problems among adolescents [26]. Adolescents in a Norwegian population study reported short sleep duration on weekdays, resulting in a sleep deficiency of approximately 2 h, and the majority of the adolescents reported sleep onset latency of more than 30 min [31]. Girls reported a higher rate of insomnia and longer sleep onset latency than boys [31]. Since sleep problems may be different for different age groups, the relationship between sleep and OCD should be studied specifically within different developmental periods, such as childhood, adolescence and adulthood, as it may differ at different developmental stages [26].

Research investigating the relationship between OCD and insomnia in adolescents and whether insomnia may be a predictor of OCD treatment outcome is very scarce, and further research is needed, particularly given the mixed and inconclusive nature of the extant findings [16, 17, 26]. Therefore, the aims of this study were to (1) estimate the prevalence of insomnia in a secondary mental health care population of adolescents meeting the criteria for OCD, (2) investigate the relationship between baseline level of insomnia symptoms and severity of OCD-symptoms, and (3) determine if baseline levels of insomnia symptoms predict treatment outcome of B4DT for OCD, while adjusting for potential confounders including depression and generalized anxiety symptoms.

## Method

### Design and participants

The adolescents in this sample were recruited from a naturalistic open trial effectiveness study approved by the Regional Committee for Medical and Health Research Ethics of Northern Norway (REK Nord: 2023-606482). The sample and procedures are the same as in Skjold et al. [34], and more details about the study are described there. The study included 43 patients, aged 16–18, from seven different specialized secondary care outpatient OCD treatment units across Norway. Two of the patients turned 18 years old some days prior to treatment, while the rest were less than 18 years old. The same standard assessment and treatment procedures were used across all of the treatment units, and these were also the procedures for this study. The standard assessment procedures included diagnostic interviews to diagnose OCD, screening for comorbidity and exclusionary diagnoses, and assessment of motivation, insight, and expectations regarding the treatment.

Patients had to be between 16 and 18 years old, diagnosed with moderate or severe OCD equivalent to a score of 16 or above on CY-BOCS, and seeking OCD treatment from the public health system in Norway to be included in this study. We used the ICD-10 criteria for diagnosing OCD, which is standard practice in public health outpatient clinics in Norway. Participants decided whether to receive the treatment after receiving information about the treatment and signing a written informed consent to participate in the study. Patients were excluded from the present study if they met the criteria for another severe disorder that needed treatment before OCD, including active psychosis, mania, active suicidality, drug addiction or a severe eating disorder with a BMI that was too low for them to be able to concentrate and complete the B4DT treatment. The participants were also excluded if they had previously been diagnosed with an autism spectrum disorder or an intellectual disability that would impair their ability to comprehend psychoeducation or manage the demands of participating in a group setting.

*Adherence and competence*: All therapists worked at specialized outpatient OCD treatment units in the public health care system. They went through courses and training in OCD treatment and the B4DT format before they were responsible for treating their own OCD patients. They also used a standardized manual for B4DT OCD treatment and worked in specialized outpatient mental health OCD units for children and adolescents.

### Diagnostics and assessment

A standard assessment procedure has been implemented across the different OCD treatment units in Norway. The standard procedure was followed in this study as well and included a prescreening conversation, a semistructured diagnostic screening interview (K-SADS-PL or MINI), the semistructured interview CY-BOCS, a checklist assessing anticipation of the treatment, and self-report measures [34]. Both the “Schedule for Affective Disorders and Schizophrenia for School-Age Children” (K-SADS-PL) and the MINI interview have good psychometric properties [35, 36] and were used to assess comorbidity and exclusion criteria for participating in the treatment. The CY-BOCS interview and self-report questionnaires measuring insomnia symptoms, general anxiety symptoms and depression symptoms were administered pretreatment, posttreatment and at the 3-month follow-up.

*Children’s Yale-Brown Obsessive Compulsive Scale (CY-BOCS)*. OCD symptoms were measured using the semistructured clinician-administered interview version of the Children’s Yale-Brown Obsessive Compulsive Scale, CYBOCS [37]. The CY-BOCS has been widely used to measure OCD symptoms, has good psychometric properties and is regarded as the gold standard clinical interview when assessing the severity of OCD symptoms [37]. The severity of obsessions and compulsions are rated based on five dimensions: time occupied by symptoms, how the symptoms interfere with daily life, distress, resistance, and control.

*Bergen Insomnia Scale.* The Bergen Insomnia Scale (BIS) is a self-report questionnaire used to measure insomnia symptoms during the past month [38]. The questionnaire consists of 6 items. Each item is rated on an 8-point Likert scale, ranging from 0 (none of the days during the week) to 7 (every day during the week). The total score ranges from 0 to 42, where higher scores indicate more severe insomnia. Items 1–3 refer to problems falling asleep, maintaining sleep, and early morning awakenings. Items 5–6 concern not feeling adequately rested, daytime impairment, and dissatisfaction with sleep. Insomnia is indicated if one or more of the variables 1–4 is rated 3 or higher, combined with a score of three or more on at least one of the variables 5–6 [38]. The BIS has shown good internal consistency and seems to be a reliable and valid instrument for insomnia, based on the DSM-IV-TR criteria for insomnia [38].

*Patient Health Questionnaire-9 (PHQ-9).* The PHQ-9 is a self-report questionnaire assessing depressive symptoms based on nine criteria in the DSM-IV, where each item is scored on a four-point Likert scale [39].

*General Anxiety Disorder-7 (GAD-7).* The GAD-7 is a self-report questionnaire measuring symptoms of general anxiety, where items are scored on a four-point Likert scale based on DSM-IV criteria [40].

### Treatment

The adolescents received the youth version of the B4DT for OCD [11]. The treatment was delivered across four consecutive days, with 3–4 patients in each group, where the patient-therapist ratio was 1:1. The parents were present on days one and four, and the treatment was an individually tailored treatment within a group format. Day one includes psychoeducation for both youths and parents, followed by separate sessions for youth exposure planning and parental guidance on reducing family accommodation for OCD. Days two and three focus on therapist-led exposure training, with brief group meetings. On day four, treatment is reviewed, emphasizing maintenance strategies and plans for exposure training over the next three weeks. The B4DT youth format is further described in the study by Riise [11].

Patients received treatment between 2020 and 2022. Due to COVID-19 regulations, some of the groups had to be delivered over video or partly over video. There were no differences in the treatment content besides these adaptations. Specifically, six patients received fully virtual video-based treatment for all four days, three patients received a hybrid version, information about COVID-19 adaptations was missing for six patients, and 26 patients received face-to-face treatment.

### Statistical analysis

We used version 27 of the IBM SPSS statistics software to perform the statistical analyses. To investigate whether insomnia symptoms prior to treatment were related to the baseline severity of OCD symptoms, we performed a Pearson correlation between scores on the Bergen Insomnia Scale (BIS) and CY-BOCS. We also ran correlations between the BIS and the other measures (GAD-7 and PHQ-9). Independent samples *t* tests and Pearson’s correlations were also used to examine the relationship between baseline characteristics such as gender, medication status, OCD duration, comorbid depression and comorbid anxiety with insomnia measured with the BIS. To investigate whether insomnia symptoms before treatment (as measured by BIS) were related to the change in symptoms of OCD (as measured by CY-BOCS), we analyzed repeated measures with linear mixed models (LMM), with time and BIS as fixed effects. The intercept was specified as random. We assumed that missing data were missing at random (MAR). LMM can handle data missing at random without imputation or listwise deletion (Enders, 2010). A diagonal covariance structure was applied, and restricted maximum likelihood estimation with the Satterthwaite approximation was chosen to estimate t- and p values. A sensitivity analysis was performed to investigate whether the effect of insomnia symptoms could be explained by baseline symptoms of generalized anxiety or depression, where we also added the GAD-7 and PHQ-9 as fixed effects to the previous analyses. In this analysis, item 3 of the PHQ-9 was removed because this item asks about sleep problems, and we would like to separate sleep problems from depressive symptoms.

## Results

All 43 patients included in the sample completed the B4DT treatment. CY-BOCS data for one patient was missing posttreatment, while CY-BOCS data for four patients were missing at the 3-month follow-up, and BIS data for five patients were missing pretreatment. We had either post- or 3-month follow-up data for all 43 patients. Demographics and a diagnostic description of the sample are summarized in Table 1. The majority of the patients (33 out of 43) were females. Only five patients reported taking psychotropic medicines, while 32 were not taking any psychotropic medicines, and the medication status was unknown for six of the patients. No patients reported taking hypnotics or melatonin.

**Table 1:** Demographics and diagnostic description of the sample (*N* = 43)

Variable	M (SD)
Age (years)	16.77 (0.53)
Duration of the disorder (years)	3.21 (2.48)
	N (%)
Gender: females	33 (76.74)
Have received previous treatment	22 (51.16)
Comorbidity Not reported	20 (46.51)
No comorbidity Comorbid anxiety Comorbid depression	9 (20.93) 11 (25.58) 7 (16.28)
Psychotropic medication No medication SSRI/SNRI Ritalin Not reported	5 (11.63) 32 (74.40) 5 (11.63) 1 (2.32) 6 (13.95)

Twenty-six of 38 patients (68.42%) who completed the BIS self-report questionnaire met the cutoff criteria for insomnia pretreatment. Pretreatment insomnia was not significantly related to gender (*p* = .140), medication status (*p* = .139), OCD duration (*p* = .129), comorbid anxiety diagnosis (*p* = .057) or comorbid depression diagnosis (*p* = .110).

Table 2 shows how the patients who scored above the cutoffs for insomnia on the BIS and below the cutoffs responded to treatment when using listwise deletion for missing data. The majority of the adolescents who scored above the BIS cutoff for insomnia were also defined as responders posttreatment (21 out of 25) and at the 3-month follow-up (20 out of 22). All patients who scored below the BIS cutoff for insomnia pretreatment were responders and in remission at the 3-month follow-up. Among the patients who scored above the BIS cutoff before treatment, two were nonresponders, and six were not in remission.

**Table 2:** B4DT OCD-treatment outcome compared with insomnia scores pretreatment

		Insomnia	No insomnia
Post	Remission	17	8
	Response	4	2
	No response	4	2
	Total (n)	25	12
3-month follow-up	Remission	16	12
	Responder	4	0
	No responder	2	0
	Total (n)	22	12

OCD-treatment outcome: Remission: Responders and a CYBOCS score ≤ 12. Response: 35% or more reduction of OCD-symptoms measured by CY-BOCS (42). Insomnia: BIS cutoffs for insomnia.

### Outcome measures

We found a moderate correlation between the baseline CY-BOCS and BIS, r [38] = 0.319, *p* = .051, that did not achieve statistical significance but could be described as a trend. In contrast, there were significant correlations between insomnia symptoms and depression and GAD symptoms. The correlation between GAD-7 and BIS pretreatment was r [38] = 0.499, *p* = .001, while the correlation between PHQ-9 without item 3 and BIS pretreatment was r [38] = 0.633, *p* < .001 (2-tailed).

Overall, patients had a significant reduction in OCD symptoms over time (F = 169.173, *p = > 0.001*), and we found that insomnia symptoms before treatment had a significant effect on CY-BOCS change over time (F = 10.365, *p* = .002). High insomnia symptom pretreatment was associated with poorer treatment outcomes. The sensitivity analyses showed no effect on change on GAD-7 (F = 2.33, *p* = .130) and PHQ-9 (F = 0.517, *p* = .474), while the effect of insomnia symptoms on change remained significant in this analysis (F = 4.66, *p* = .033) while controlling for GAD-7 and PHQ-9.

## Discussion

In this study, we found a high prevalence of insomnia in adolescents with OCD. 68% of the patients scored above the cutoffs for insomnia using the BIS cutoff. This is higher than the prevalence among adolescents in general [31] but comparable to the results of sleep problems for youth OCD patients in the NordLOTS study [16]. Since this study used other criteria and methods to assess sleep problems and included a wider range of sleep problems in addition to insomnia and a wider age range, there are some challenges in comparing our results with this study [16]. The high prevalence of insomnia in this sample could be a reminder for clinicians to assess for and be aware of insomnia symptoms as well as OCD among adolescent OCD patients.

Furthermore, OCD symptoms and insomnia before treatment were not significantly correlated, while insomnia symptoms before treatment correlated with symptoms of general anxiety and depression symptoms before treatment.

We did find that insomnia symptoms before treatment were significantly related to treatment outcome, measured by a change in CY-BOCS symptoms across time. This is in line with the results reported in the NordLOTS study of the association between sleep problems and OCD with a child and adolescent sample [15]. Our results suggest that insomnia was a predictor of treatment outcome in our sample, even when controlling for general anxiety symptoms and depressive symptoms. Overall response and remission rates were very high for this sample. This is in line with the results from several previous studies on the B4DT [8, 12, 34]. Clinically, it is challenging to draw firm conclusions regarding the treatment implications of this finding, and it is important to note that most of the adolescents in this sample who scored above the cutoffs for insomnia still responded well to the treatment. Information about sleep difficulties should be assessed and monitored as a risk factor for nonresponse, but the results from this study do not imply that insomnia is a consistent roadblock that has to be solved to profit from B4DT for OCD. Sleep problems may explain smaller variations in the degree of treatment effect, rather than the binary distinction between response and nonresponse.

Further research is needed to see if the results are replicated and to look closer into potential mechanisms that might link insomnia and OCD. It would be interesting to see if treating insomnia in addition to the B4DT for OCD either before, during or after the B4DT would make a difference in treatment outcome, functioning and quality of life for the adolescents who meet criteria for both diagnoses.

The results from this study differ from the studies of insomnia and OCD-treatment outcomes for adults receiving the B4DT, since insomnia did not significantly predict treatment outcome in the adult sample [18, 19]. However, sleep problems and needs may differ at different developmental stages during life [26], so the association between insomnia and OCD treatment outcomes may not be the same for children, adolescents and adults. Different environmental factors that affect sleep in different age groups, chronotype changes, different levels of maturity, emotional control, and inhibition at different developmental stages in life could contribute to those differences.

Several mechanisms that may have an effect on OCD treatment outcomes can be affected by insomnia. For example, sleep has been found to be an important predictor of fear extinction learning [23], which some have proposed to be involved in exposure-based therapies. In addition, sleep and alertness can affect attention and inhibitory control mechanisms, making it more difficult to resist compulsive rituals when very tired [22–26]. Reduced attention or learning retention due to insomnia could make it harder to learn all the treatment principles and the new ways of dealing with anxiousness and obsessions, while doing the exposure tasks with full response prevention could be harder if insomnia reduced extinction, inhibition, and the ability to hold back and stop doing rituals. People may also be less motivated to work hard and put in less effort in their work when tired [41]. Since the B4DT requires effort from the patients, as they work on applying the treatment principles in their lives, this could also affect OCD treatment outcomes. Thus, insomnia may not only be a predictor of the treatment outcome but could also be a moderator.

Importantly, we utilized the published cutoff scores for insomnia on the BIS, which are based on the DSM-IV criteria for insomnia rather than DSM-5, and tend to inflate the prevalence numbers somewhat, and the number of patients with insomnia would be somewhat lower using the DSM-5 criteria, as these criteria are stricter [31]. There may also be other aspects of the BIS that may inflate the prevalence numbers, such as severity of symptoms rated as number of nights with insomnia problems, rather than how severe the problems were each specific night or during that past week. The criteria used to define insomnia differ in different studies, and the assessment tools also differ [26, 31], making it harder to compare the studies. It would be beneficial for the field if there was a consensus about definitions and which measurement tools should be used across different studies.

Other potential study limitations should also be noted. In this study, insomnia and sleep problems were only measured by BIS and not a diagnosis of insomnia disorder based on a clinical interview. BIS does not measure circadian rhythm problems or organic sleep disorders such as restless legs and sleep apnea, and our assessment did not include any questions about these disorders. Circadian rhythm problems are common among youths, and recent studies have found that this is also more common among OCD patients than controls [28, 42, 43]. Thus, our research on insomnia lacks information about other relevant sleep problems that may be especially relevant and common among adolescents with OCD. Further research should include circadian rhythms when assessing the relationship between sleep and OCD among adolescents.

Our sample includes many more females than men, and research about gender differences in insomnia among adolescents suggests that more adolescent girls struggle with insomnia than boys [31]. Thus, our results could have been different if our sample was more balanced, consisting of more boys.

Our study was a naturalistic study with a limited sample size, studying adolescents seeking help at specialized OCD-treatment units in Norway, not a randomized controlled trial. Thus, we cannot control for unknown confounding third variables that may have affected the results, including therapist education and experience. We do not know how many patients were assessed for OCD, and then excluded from the treatment.

Further research should include larger samples and see if the results are replicated with other age groups, in addition to 16-18-year-olds, to determine the effect of insomnia at different developmental stages. Several studies suggest that OCD and insomnia may have a bidirectional relationship [16, 26], but we have not been able to look at this in the current study, as we did not have enough data about insomnia after treatment. Further research could also aim to combine subjective sleep measures with objective measures of sleep quality, allowing for a more comprehensive understanding of sleep quality. Future research should also examine confounding variables, particularly ADHD. With its high prevalence in adolescents with OCD and strong association with sleep problems, ADHD may significantly influence both OCD symptoms and sleep disturbances.

## Conclusion

This research indicates that many adolescents with OCD have insomnia, and that insomnia and OCD symptoms are related, although exactly how they are related remains unknown. Even though the treatment was effective for the majority of the adolescents with OCD and insomnia in this study, insomnia symptoms before treatment did predict the treatment outcome in this sample, measured by changes in OCD symptoms from before treatment to after treatment.

We cannot make any causal conclusions about the findings, but they may have implications for treatment. This study is a reminder of the relevance of including insomnia symptoms while assessing adolescents with OCD. If an adolescent OCD patient is struggling and has a poor treatment outcome, therapists should be aware of the patient’s insomnia status and consider insomnia treatment interventions. The sample in this study is quite small, with only 43 participants, and the study has its limitations. Further research is needed to determine whether insomnia pretreatment predicts treatment outcomes with larger samples and other age ranges while also controlling for additional variables. In addition, research on possible mechanisms contributing to the association between OCD and insomnia is needed, as knowledge of mechanisms could lead to treatment refinement. It would be interesting to determine if treating both insomnia and OCD for those who meet the criteria for both diagnoses would lead to better OCD treatment outcomes, better functioning and quality of life.

## Data Availability

The anonymized datasets used during the current study are available from the corresponding author upon reasonable request.

## List of Abbreviations

OCD: Obsessive-Compulsive Disorder
CBT: Cognitive behavioral therapy
ERP: Exposure and response prevention
B4DT: Bergen 4-Day Treatment
CY-BOCS: Children’s Yale-Brown Obsessive Compulsive Scale
BIS: Bergen Insomnia Scale
GAD-7: General Anxiety Disorder-7 (self-report questionnaire)
PHQ-9: Patient Health Questionnaire
K-SADS-PL: Schedule for affective disorders and schizophrenia for school-age children-present and lifetime version
MINI: The Mini International Neuropsychiatric Interview
REK: Regional Committees for Medical and Health Research Ethics
DSM IV/V: Diagnostical and Statistical Manual of Mental Disorder (4th/5th edition)
SSRI: Selective serotonin reuptake inhibitors
SNRI: Serotonin and norepinephrine reuptake inhibitors
